# Learning a reach trajectory based on binary reward feedback

**DOI:** 10.1038/s41598-020-80155-x

**Published:** 2021-01-29

**Authors:** Katinka van der Kooij, Nina M. van Mastrigt, Emily M. Crowe, Jeroen B. J. Smeets

**Affiliations:** Department of Human Movement Sciences, Vrije Universiteit Amsterdam, 1081 BT Amsterdam, The Netherlands

**Keywords:** Learning and memory, Motor control

## Abstract

Binary reward feedback on movement success is sufficient for learning some simple sensorimotor mappings in a reaching task, but not for some other tasks in which multiple kinematic factors contribute to performance. The critical condition for learning in more complex tasks remains unclear. Here, we investigate whether reward-based motor learning is possible in a multi-dimensional trajectory matching task and whether simplifying the task by providing feedback on one factor at a time (‘factorized feedback’) can improve learning. In two experiments, participants performed a trajectory matching task in which learning was measured as a reduction in the error. In Experiment 1, participants matched a straight trajectory slanted in depth. We factorized the task by providing feedback on the slant error, the length error, or on their composite. In Experiment 2, participants matched a curved trajectory, also slanted in depth. In this experiment, we factorized the feedback by providing feedback on the slant error, the curvature error, or on the integral difference between the matched and target trajectory. In Experiment 1, there was anecdotal evidence that participants learnt the multidimensional task. Factorization did not improve learning. In Experiment 2, there was anecdotal evidence the multidimensional task could not be learnt. We conclude that, within a complexity range, multiple kinematic factors can be learnt in parallel.

## Introduction

In the backyard, Katy practices a dance move to imitate a video clip. She doesn’t seem to improve. What would you do to help? Probably you’ll provide her with feedback such as telling her which attempts are better than others. Each time you tell her an attempt is successful, a reward signal is delivered to Katy’s brain. This signal can form the basis for a type of motor learning called reward-based motor learning, also called ‘reinforcement learning’ or ‘operant conditioning’^1,2^. Such learning relies on a combination of trial-by-trial variations in movements (‘exploration’) and repetition of successful movements (‘exploitation’)^1,3–7^. Reward-based learning does not scale well to multidimensional problems^8,9^ in which many factors may underlie the reward because binary reward feedback is sparse. Since even a simple movement such as a dance move is composed of multiple kinematic factors (e.g. direction and curvature), an open question is how reward-based learning contributes to motor learning. In this study, we examine the influence of task complexity on reward-based motor learning.

Reward-based motor learning has mainly been demonstrated in one-dimensional tasks in which reward is based on a single factor of the movement. A common task is the centre-out reaching task in a horizontal plane^1,4–6,10,11^. In this task, participants make centre-out ‘shooting’ movements through targets in different directions while the relationship between direction and visual feedback is perturbed. Success is determined by a 1D factor: the movement direction. Whether reward-based motor learning is possible in multidimensional tasks is unclear. We previously showed that learning of a visuomotor perturbation in a three-dimensional pointing task was not possible when participants received reward feedback based on a three-dimensional position error^12–14^, whereas, in a one-dimensional version of the task, learning was possible^14^. Manley et al.^15^ showed that reward-based learning of a visuomotor perturbation in a multidimensional task was only possible when participants were aware of the task-relevant dimension. However, there is also evidence that reward-based learning of a multidimensional task is possible. In a reaching task, in which the reward depended on the relative contribution of the wrist and elbow to the movement, the joint configuration used shifted towards the rewarded configuration^16^. In a walking task, a new walking pattern could be learnt based on reward feedback^17^.

The evidence that task-complexity hampers reward-based motor learning primarily comes from studies that impose a visuomotor perturbation (i.e. reaching off-target to see the cursor hit the target; for a review see Ref.^18^ to study learning. Learning such a perturbation might be different from learning to correct for the natural visuomotor biases^19,20^. One reason why learning a perturbation might be more difficult is that humans might use prior information on which dimension is the task-relevant one^8^. When a perturbation is imposed, prior experience does not provide useful information about task relevance. The influence of task complexity on reward-based motor learning thus still needs to be established in a task that does not involve the perturbation of feedback.

If task-complexity hampers reward-based motor learning, training might benefit from simplifying the task. It has been proposed that humans engage in ‘representation learning’ to solve the dimensionality problem: focusing on one dimension at a time to determine whether that dimension is the relevant dimension^8^. This process might be solved for the participant by factorizing the feedback in a multidimensional task: giving feedback on each factor in a separate phase of the training. Would Katy’s dance move improve more if she receives feedback on her the angle of her movement first, followed by the curvature, or does it improve more if she receives feedback on both simultaneously? Here, we investigated the effect of feedback factorization on learning a multidimensional task with binary reward feedback. We asked whether reward-based motor learning is possible in multidimensional tasks and further asked whether learning of a factor depends on the complexity of the task. That is: is it more difficult to learn the direction of a dance move when also practicing the curvature? We also examined whether learning of the multidimensional task is improved if the feedback is factorized.

We address these questions in a three-dimensional trajectory matching task akin to trajectory learning tasks used in other studies on reward-based motor learning^7,21^. We asked participants to copy a simple trajectory by moving the unseen hand in virtual reality. In Experiment 1 they copied a slanted line and in Experiment 2 they copied a curved line. Without training, participants make systematic errors in this task, and the purpose of the feedback was to reduce these errors. In Experiment 1 we factorized the task into a slant and length factor and compared learning from factorized feedback to learning from the composite of these factors. In Experiment 2, we factorized the task into a slant and curvature factor and compared learning from factorized feedback to learning from feedback on the total deviation between the target and drawn trajectory: the integrated error.

## Experiment 1

In Experiment 1, we studied the learning of two factors of a slanted line: slant and length. The experiment consisted of three learning phases with binary performance feedback and a baseline and retention phase in which no performance feedback was provided. To test whether learning improves by factorizing feedback, we divided the participants into three groups with a different order of feedback during the three learning phases of the experiment. We predict that the groups who receive factorized feedback (i.e. Length First and Slant First group) improve more on the multidimensional task than the group who did not receive factorized feedback (Composite Always group). Furthermore, we predict that reducing the complexity of the task by factorizing feedback improves the learning of a single factor.

## Results Experiment 1

**Figure 1:**
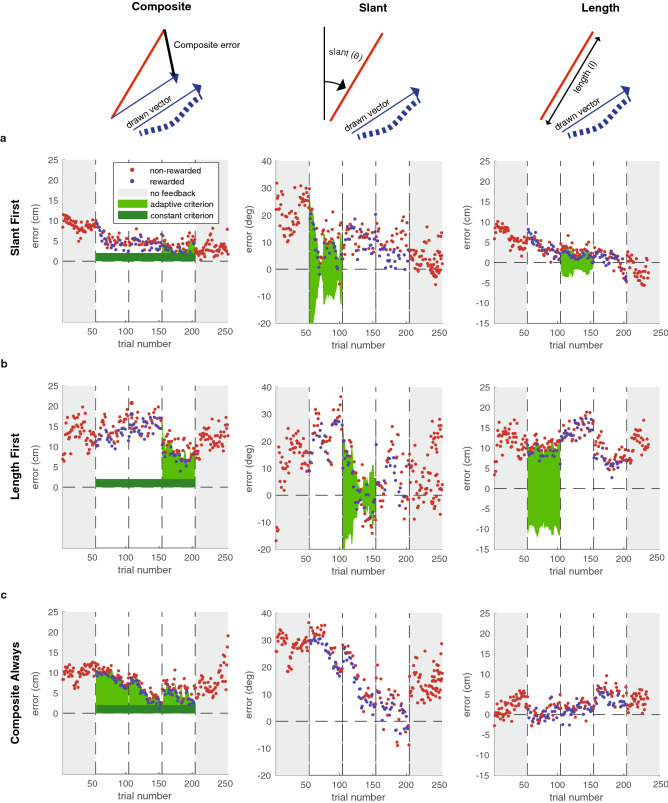
Time course of Experiment 1. (**a–c**) The errors in individual trials for three example participants. The green shaded areas represent the reward criteria that were applied in each phase.

In virtual reality, participants viewed a line slanted in the sagittal plane (vertical along the participant’s line of sight; see “Methods” for a detailed description of the task), remembered this line, and copied it with an invisible handheld controller. They knew that the feedback would be based on slant, length, or the composite. In the three learning phases, binary reward feedback was provided. When a trial was rewarded, the target line coloured green, and 5 points were added to a cumulative score that was displayed in virtual reality. When a trial was not rewarded, the target did not change colour, and no points were scored. We used an adaptive reward criterion (see “Methods”) which was dependent on the history of errors. The green areas in Fig. 1 show the resulting reward criteria for an example participant in each different group.

**Figure 2:**
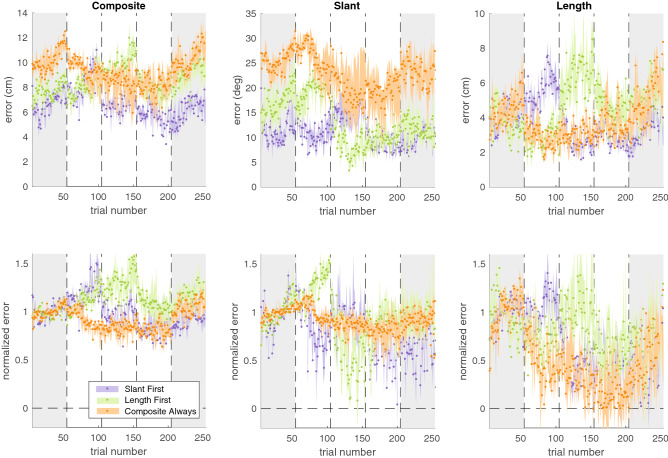
Median time course of errors across groups. Top row: absolute errors. Bottom row: normalized errors. Shaded errors represent the bootstrapped 95% confidence interval of the median.

We studied how much participants learnt based on how well they were able to reduce their errors in the slant factor, the length factor, and the composite. Figure 2 shows the median time course of errors for each group. To analyse how learning depended on the factorization of feedback, we determined the *error level* for each phase: the absolute median error in the last 20 trials of that phase. The median error levels for slant, length and, the composite were 16.1°, 4.0 cm, and 8.3 cm respectively (Fig. 2a). Learning was defined as the error level in the baseline phase minus the error level in a specific learning phase (i.e. Baseline—Learning phase 1; Baseline—Learning phase 2; Baseline—Learning phase 3).

**Figure 3:**
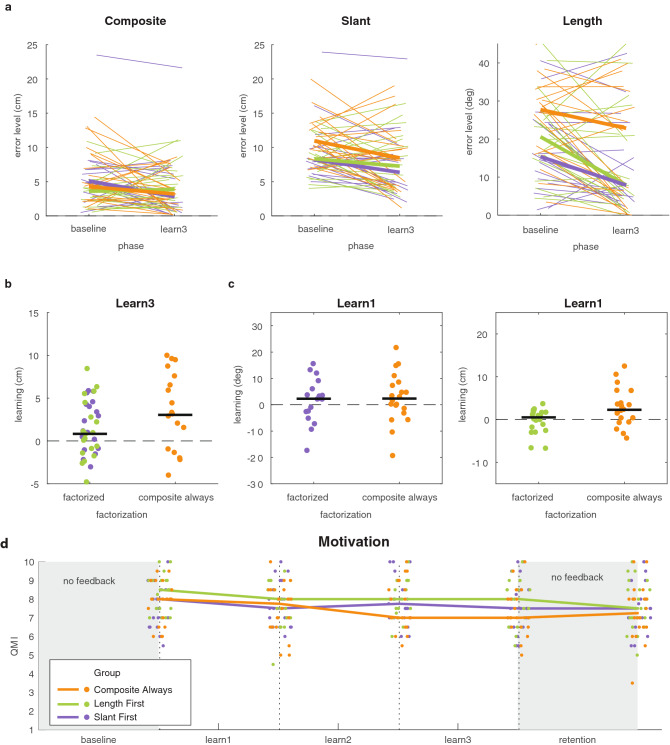
Results on learning in Experiment 1. (**a**) Error level in the baseline phase and the third learning phase for the multidimensional task and the chosen factors. Thin lines represent individual participants whereas the thick lines represent the group median. (**b**) Learning of the multidimensional task in the third learning phase for the groups that received factorized feedback and the group that received composite feedback. (**c**) Learning of slant and length in the first learning phase. Dots represent individual participants and the black lines represents the median across participants. (**d**) Motivation in the three groups as a function of phase number, measured with the Quick Motivation Index that asked about enjoyment and motivation to continue (QMI; van der Kooij et al., 2019). Dots indicate the individual participants; the lines connect the median of each group. For each participant, the horizontal position of the data is the same across all panels.

Since motivation may influence how well participants learn from reward feedback (Holland et al., 2019), we asked participants about their motivation following each phase using a Quick Motivation Index (QMI^22^) The three groups reported similar motivation in all three phases (Fig. 3d), suggesting that possible between-group differences in learning are unlikely to be the consequence of differences in motivation.

Since we tested adaptation to naturally occurring biases, these biases differed between individuals. We had no hypotheses on differences between groups. However, the absolute errors in Fig. 2 show considerably larger baseline biases for the Composite Always group than for the other groups however. To answer a reviewer’s question, we tested whether there were differences between groups at baseline. We compared the biases in slant, length and in the composite error between groups using one Kruskal–Wallis test for each factor. We found that the composite error at baseline differed significantly between groups (*X*^2^ = 6.43, *p* = 0.04) although the biases in the individual factors did not differ significantly between groups (for slant: *X*^2^ = 5.81, *p* = 0.05; for length: *X*^2^ = 4.28, *p* = 0.11).

A prerequisite for factorization to benefit learning is that errors in the individual factors can be reduced. We therefore first performed an across-groups analysis in which we tested whether errors in the slant factor, the length factor, and the composite were reduced across the entire task (Fig. 3a). To do so, we tested whether the error level in the baseline phase was larger than the error level in the third learning phase using three one-sided Wilcoxon signed-ranks tests, testing the *p*-value against a Bonferroni-corrected alpha of 0.02. In addition, we used the Bayesian equivalents of the tests for a more fine-grained classification of the evidence. We interpret the Bayes factors according to Jeffreys evidence categories^23^ such that *BF* = 1 is absence of evidence 1 < *BF* < 3 is anecdotal evidence for the hypothesis, BF > 3 is moderate evidence for the hypothesis and 0. 3 < *BF* < 1 is anecdotal evidence for the null hypothesis and *BF* < 0.3 is moderate evidence for the null hypothesis.

We found that all errors were closer to zero by the third learning phase: the slant error was reduced (*z* = 3.38, *p* < 0.01, *BF*_+0_ = 1.78, *δ* = 0.65, 95% CI [0.05, 1.6]); the length factor was reduced (*z* = 2.12, *p* = 0.02, *BF*_+0_ = 1.91, *δ* = 0.49 95% CI [0.02, 1.39]); and the composite error was also reduced (*z* = 2.86, *p* < 0.01, *BF*_+0_ = 1.38, δ = 0.57, 95% CI [0.04, 1.51]). We take this as anecdotal evidence that the multidimensional task and the slant factor could be learnt (1 < *BF*_+0_ < 3).

To test the prediction that learning the multidimensional task would be improved by providing factorized feedback, we compared learning between the two factorized groups and the Composite Always group (Fig. 3b). We did so for learning at the end of the third learning phase in which all groups had received feedback on the multidimensional task. We used a one-sided Mann–Whitney U test to assess whether the factorized groups (Slant First group; Length First group) had learnt more than the Composite Always group. We found that participants who received factorized feedback did not learn more than participants who received composite feedback (*z* = − 1.09, *p* = 0.86, *BF*_+0_ = 0.2, δ = 0.13, 95% CI [0.01, 0.49]). We take this as moderate evidence that factorization did not improve learning (0.1 > *BF*_+0_ < 0.3).

To test the prediction that learning of a factor was improved by reducing task complexity, we compared the factorized groups to the Composite Always group. We did so for learning at the end of the first learning phase (Fig. 2c). In this phase, the factorized groups experienced a less complex and one-dimensional task than the Composite Always group who performed a multidimensional task. Two one-sided Mann–Whitney U tests, one for slant and one for length, with a Bonferroni-corrected alpha of 0.02 showed that, for both the slant factor and the length factor, learning was not improved when they were the only performance-relevant factor (Slant: *z* = − 0.22, *p* = 0.58, *BF*_+0_ = 0.13, δ = 0.1, 95% CI [0.01, 0.41]; length: *z* = − 2.28, *p* = 0.99, *BF*_+0_ = 0.11, δ = 0.08, 95% CI [0.01, 0.35]). Hence, we found moderate evidence that reducing task complexity by factorizing the task did not improve learning (0.1 > *BF*_+0_ < 0.3).

### Slant first implicit group

The finding that participants could learn the multidimensional task based on binary reward feedback evoked the question of whether explicit information on the rewarded factors was necessary to learn from factorized feedback. If learning occurred on an explicit level^11,24–26^ or required the participant to reduce dimensionality by some form of factorization^8,27^, the participant would have had more factors to consider when no explicit information on the rewarded factors was provided. For instance, because in addition to slant and length, the smoothness of the trajectory was considered. Therefore, we would expect learning to be more difficult without explicit information on the rewarded factors.

To test the necessity of explicit information on which factors were rewarded to learn from factorized feedback, we measured an additional group: The Slant First Implicit group. The experiment was identical to that for the Slant First group, except for the instruction that participants received. Participants were only told that when the target coloured green and points were scored they had performed “well”. They received no information on the error that the feedback was based on.

**Figure 4:**
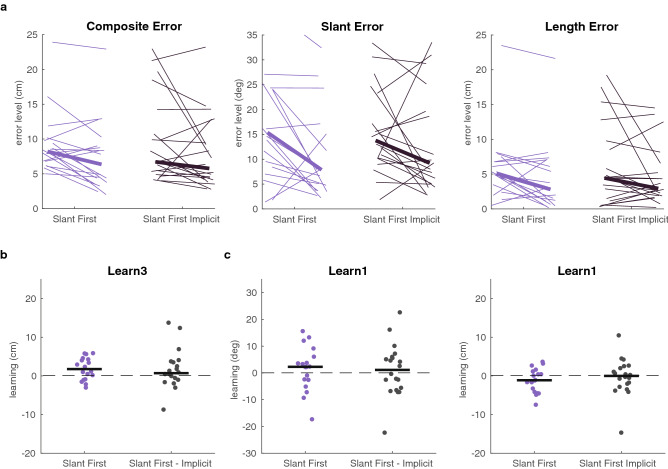
Learning with explicit or implicit feedback on which factor was rewarded. (**a**) Error level in the baseline phase and in the third learning phase for the Slant First group and for the Slant First Implicit group. Thin lines connect for individual participants the error level in the baseline (left) and in the third learning phase (right). Thick lines represent the median for each group. (**b**) Learning of the multidimensional task in the third learning phase. Dots represent individual participants and the black line represents the median across participants. (**c**) Learning of the slant factor and length factor in the first learning phase. Dots represent individual participants and the black line represents the median across participants. For each participant, the horizontal position of the data is the same across all panels.

To test whether participants could learn the factors and the composite, we tested whether the slant error, length error, and composite error were closer to zero in the third learning phase than in the baseline phase (Fig. 4a). We used three right-tailed Wilcoxon rank-sum tests and *p*-values were compared against a Bonferroni-corrected alpha of 0.02. In this experiment we found no significant reduction of the composite error (*z* = 1.51, *p* = 0.06, *BF*_+0_ = 1.14, δ = 0.53, 95% CI [0.03, 1.52]), the slant error (*z* = 1.18, *p* = 0.12, *BF*_+0_ = 0.9, δ = 0.45, 95% CI [0.02, 1.39]), or the length error (length: *z* = 0.73, *p* = 0.23, *BF*_+0_ = 0.81, δ = 0.43, 95% CI [0.02, 1.35]). There were no differences between the implicit and explicit groups: neither in learning the multidimensional task (Fig. 4b: *z* = 0.51, *p* = 0.30; *BF*_10_ = 0.34, CI [− 0.47, 0.67]), nor in learning of slant or length by the first learning phase (Fig. 4c; for slant: *z* = 0.04, *p* = 0.48, *BF*_10_ = 0.32 [CI − 0.57, 0.56]; for length: *z* = − 0.89, *p* = 0.81, *BF*_10_ = 0.44, CI [− 0.84, 0.32]).

## Experiment 2

**Figure 5:**
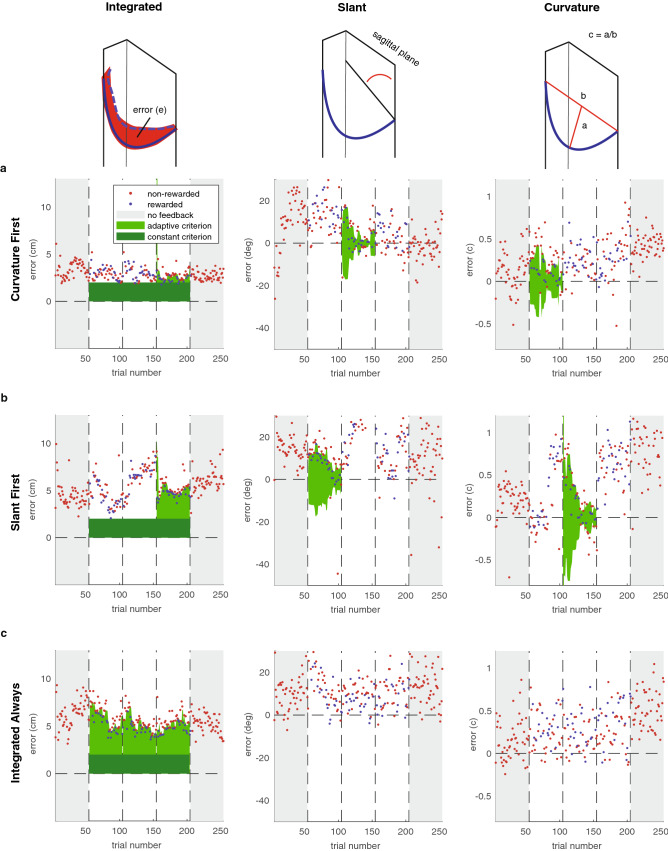
Time-course of Experiment 2. (**a**) Example participant ‘Curvature First’ group. (**b**) Example participant ‘Slant First’ group. (**c**) Example participant ‘Integrated Always’ group. (**a–c**) Left panel: integrated error; middle panel: slant error; right panel: curvature error. The green shaded areas represent the reward criteria that were applied in each phase.

The results from Experiment 1 showed that two factors of a reach trajectory (slant and length) can be learnt based on binary reward feedback. In Experiment 2, we increased the complexity of the multidimensional task by providing feedback on the entire movement (the integrated error) rather than on two factors of the movement and by providing a curved target trajectory (see “Methods” for a detailed description of the task). Hence, besides slant and length, also curvature, smoothness and distance contributed to the rewarded performance. We factorized the task by providing feedback on a slant factor, a curvature factor, or on the entire movement (integrated error, Fig. 5) and participants were informed of this. Two groups received factorized feedback. A ‘Slant First’ group received feedback on slant in their first learning phase, feedback on curvature in their second learning phase, and ended with feedback on the integrated error. A ‘Curvature First’ group received feedback on curvature in their first learning phase, feedback on slant in their second learning phase, and ended with feedback on the integrated error. The ‘Integrated Always’ group received feedback on the integrated error throughout the entire learning phase.

## Results Experiment 2

**Figure 6:**
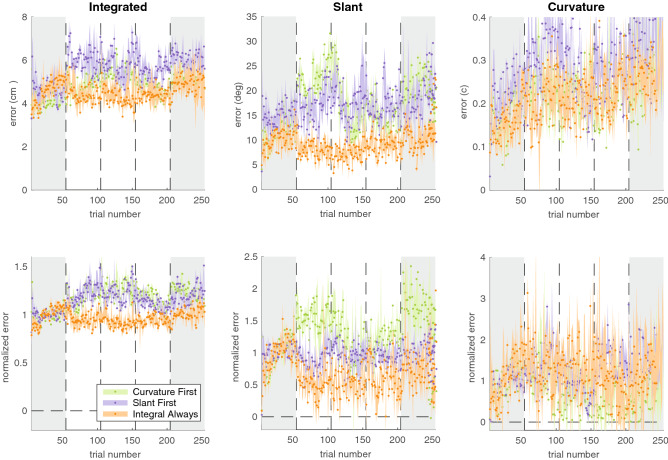
Median time course of errors across groups. Top row: absolute errors. Bottom row: normalized errors. Shaded errors represent the bootstrapped 95% confidence interval of the median.

As in Experiment 1, the time course of errors showed differences between groups (Fig. 6); the differences at baseline seemed smaller than in Experiment 1. We again analysed differences in learning the different factors based on the error level in the last 20 trials of a phase.

To test whether there were differences between groups at baseline we compared the biases in slant, length, and in the composite error between groups using one Kruskal–Wallis test for each factor. We found that there were no significant differences between groups at baseline (for slant: *X*^2^ = 2.03, *p* = 0.36; for curvature: *X*^2^ = 2.97, *p* = 0.22 for the integral error: *X*^2^ = 2.13, *p* = 0.35).

**Figure 7:**
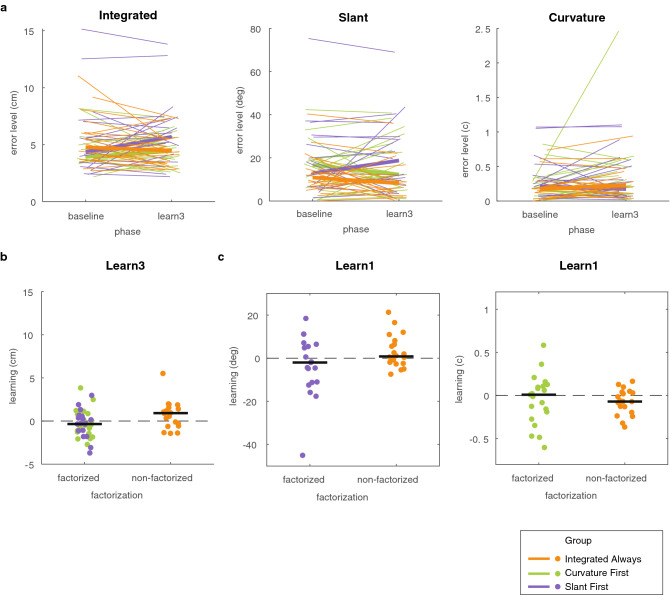
Results on learning in Experiment 2. (**a**) Error level in the baseline phase and the third learning phase for the three different groups. Thin lines represent individual participants whereas the thick lines represent the group median. (**b**) Learning of the multidimensional task in the third learning phase. (**c**) Learning of the slant (left panel) and curvature (right panel) factor in the first learning phase. Dots represent individual participants and the black line represents the median across participants. Dots represent individual participants and the black line represents the median across participants. All subfigures use the same scatter in the x-position of the dots.

As in Experiment 1, we started with an across-groups analysis in which we tested whether the error in the different factors was reduced across the entire task. To do so, we tested whether the error level in the baseline phase was larger compared to the error level in the third learning phase using three one-sided Wilcoxon signed-ranks tests (Fig. 7a). We tested *p*-values against a Bonferroni-corrected alpha of 0.02. We found that none of the errors were reduced by the third learning phase: the slant error wasn’t reduced (*z* = 0.29, *p* = 0.38, *BF*_+0_ = 0.6, δ = 0.34, 95% CI [0.02, 1.14]); the curvature error wasn’t reduced (*z* = − 2.14, *p* = 0.98, *BF*_+0_ = 0.34, δ = 0.24, 95% CI [0.01, 0.93]) and the integral error wasn’t reduced (*z* = − 0.03, *p* = 0.51, *BF*_+0_ = 0.6, δ = 0.33, 95% CI [0.01, 1.3]). We take this as anecdotal evidence that the multidimensional task and the individual factors could not be learnt (0.3 < *BF*_+0_ < 1). Perhaps, the attentive reader notices the difference between the Integral Always and factorized groups in the three learning phases in Fig. 6. We would like to point out that this might a motivational effect rather than a learning effect. As the ability to learn the chosen factors individually is a prerequisite for factorized feedback to benefit learning, we refrain from testing the prediction that factorization benefits learning of the multidimensional task. Instead, we show the data on learning of the multidimensional task and the individual factors in Fig. 7a,b).

## Discussion

In this study, we investigated reward-based motor learning of a more complex target movement than the centre-out reaching movements that are generally used (e.g. Refs.^1,4,5^). Rather than basing feedback on reach angle, we based feedback on multiple kinematic factors of the movement trajectory. We tested whether participants could learn the task based on binary reward feedback and whether learning could be improved by reducing task complexity. We did this by providing factorized feedback, focusing on only one factor of a multidimensional task at a time. We addressed these questions in a trajectory matching task that participants performed in three-dimensional virtual reality and that allowed us to test learning without the use of any perturbation to introduce errors. In Experiment 1, there was anecdotal evidence that the multidimensional task could be learnt: the composite error was reduced. Factorization did not benefit learning. In Experiment 2, there was anecdotal evidence that the more complex multidimensional task could not be learnt.

Bayesian analyses showed that our study was limited in providing only weak evidence that at best can be classified as ‘anecdotal’, which has also been referred to as ‘absence of evidence’^23^. When designing our study, we used the same sample size as in our studies on reward-based motor learning in a visuomotor adaptation paradigm (e.g. Ref.^14^). Power issues with the current study highlight that we should have better considered the required sample size when studying how participants reduce naturally occurring biases that vary between participants. Despite these power issues, we do believe that our study provides some insight in reward-based motor learning of a multidimensional task, especially those planning to set up a study in this area. Below we discuss the main results in detail.

Our main question was regarding factorization. Why did factorization not improve learning of the multidimensional task in Experiment 1? It might be that humans learn the factors of a multidimensional task in parallel^8^ rather than serializing the task as has been proposed in the literature^28^. On the other hand, we might not have designed our study properly to observe a benefit of factorization. First, a prerequisite for factorization to improve learning is that the chosen factors can be learnt. Possibly, we used too few trials to find a benefit of factorization. Second, the composite group might have learnt more because this group showed larger errors at baseline. Third, we based reward feedback on a combination of an adaptive and constant criterion. The constant criterion rewarded near-perfect performance and was based on the multidimensional task whereas the adaptive criterion depended on recent performance and could be based on the individual factors. Learning of the individual factors might have been greater if the constant component had been based on the chosen factor as well.

We found anecdotal evidence that the multidimensional task in Experiment 1 could be learnt whereas there was anecdotal evidence that the multidimensional task in Experiment 2 could not be learnt. The difference in results can be attributed to Experiment 2 using a more complex task in which the entire trajectory instead of the composite of two factors (as in Experiment 1) should be matched. Hence, besides length and slant, also curvature, smoothness and position affected the performance feedback. In the introduction, we mentioned that results on reward-based motor learning in multidimensional tasks are mixed and these results add to this. Overall, it seems that some complex tasks can be learnt while there is a limit to the complexity that allows for reward-based learning. One-dimensional tasks are generally learnt^1,4–6,14^. Two-dimensional tasks also seem to be learnt^7,17,21^ whereas a 3D dimensional task could not be learnt^12,14^. Our results fit well with this pattern of multidimensional tasks being learnt until a certain complexity is reached. The task in Experiment 1, which was learnt, could be considered a two-dimensional task whereas the task in Experiment 2, which was not learnt, had additional dimensions of curvature, lateral slant and smoothness.

Several learning mechanisms could be conceived that are capable of dealing with multidimensional tasks. On the one hand, learning might be factorized in different learning processes, called a “conquer and divide” strategy^27^. Task-relevance might be determined by a “gating module” that supervises the outcome of the different learning processes and assigns weights to their outcomes based on task-relevance^27^. In addition to factorization, or as an alternative strategy, humans might have prior hypotheses on the task-relevant dimension and simplify the task by gating attention towards those task-relevant dimension^8^. Considering the underlying learning mechanism, it is interesting that performance seemed to drift back to the baseline in the no-feedback phase (Figs. 3, 6). Such poor retention has also been observed in another study in which participants were learning to compensate for natural biases^20^, in contrast with the good retention reported in several studies that used a visuomotor rotation task^1,5,29^. It might be that poor retention was due to learning being driven by an explicit process. The idea that learning is driven by an explicit process has been proposed several times in the context of reward-based motor learning^11,25,26^.

In general, there may be limitations to *which* set of factors can be learnt together. The kinematic factors that we used—slant and length—are related because both depend on depth perception. Learning these two factors in parallel may be entirely different from learning two less related factors or learning the components of a sequence. On the one hand, separated factors might be difficult to learn together because they are not naturally explored and exploited together. For instance, in a visuomotor rotation task, the influence of the number of targets on learning depended negatively on the spatial separation between the targets^30^. On the other hand, separated factors might be *easy* to learn together because they are naturally factorized, which might facilitate the credit assignment problem. In this realm, an important question is to what extent and how the learning mechanism is factorized. If learning is not factorized, the movement trajectory is explored and reinforced as a single unit. On the neural level, this could be associated with the exploration and reinforcement of the entire neural population involved in a movement^31^. If learning is compartmentalized, the subdivision might occur on many levels: one based on effectors^9^ or muscle synergies^32^, one based on kinematic and perceptual factors, and one based on separation in time.

To conclude, binary reward feedback was sufficient to learn to reduce naturally occurring errors in one multidimensional, yet still relatively simple trajectory-matching tasks. There was no evidence that reducing task complexity by factorizing feedback improved learning of the separate factors (i.e. slant, length or curvature) when they were the only factor to be learnt, or that reducing task complexity improved learning of the multidimensional task.

## General methods

### Participants

In total 137 students from the Vrije Universiteit Amsterdam participated in the study. All participants had stereovision acuity greater than 60″ (assessed using the StereoFly test). Ethical approval for the study was provided by the local ethical committee (VCWE) of the Vrije Universiteit Amsterdam in accordance with the declaration of Helsinki. Participants provided informed consent before participating in the study.

### Set-up

**Figure 8:**
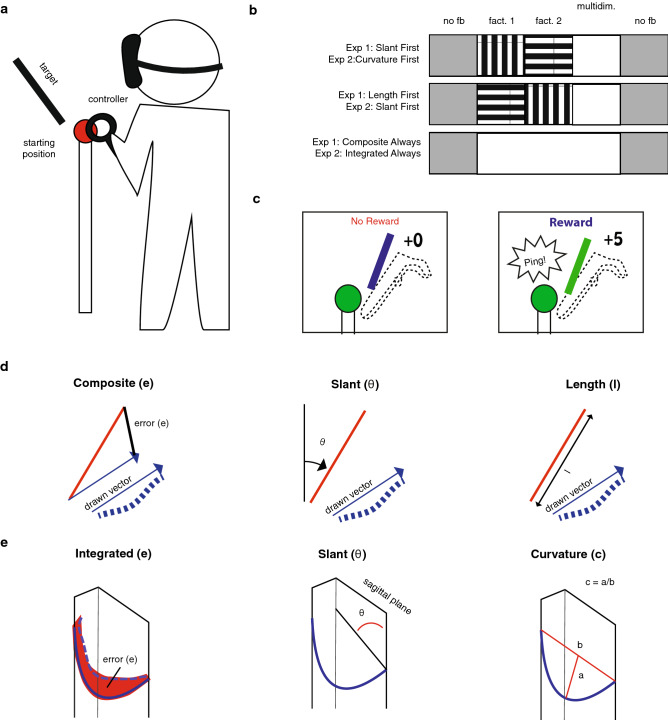
General methods. (**a**) Cartoon illustration of the set-up, target, starting position, and participant (not drawn to scale). (**b**) Order of feedback for the different groups in the different phases of the experiment (baseline, learn1, learn2, learn3, and retention phase). Striped blocks represent phases in which the participant received feedback on a single factor. (**c**) Binary reward feedback. (**d**) Target trajectory (red), actual trajectory (dashed blue curve), and factors used in Experiment 1. (**e**) Target trajectory (continuous curve), and factors used in Experiment 2.

We used an HTC Vive virtual reality system to generate stimuli and record movements. The movement task was performed with a controller that participants held in their dominant hand. We simulated a simple virtual environment in which participants were positioned behind a virtual pole with a red ‘starting sphere’ (diameter 6 cm) on top (Fig. 8a). The visual target was a 1 cm wide line, the shape and position of which depended on the experiment. To facilitate moving towards the starting sphere, a white 5 cm diameter ball could provide feedback of hand position. When the white sphere disappeared in the starting sphere, participants knew that they had reached the required position. The trial number and the cumulative score were continuously displayed above the target.

### Task

Reward-based motor learning was assessed in a three-dimensional trajectory-matching task in which participants were asked to copy a remembered line by moving a hand-held controller. To start a trial, participants had to align the controller with the starting sphere (Fig. 8a). To help participants achieve this, visual feedback on the controller position was given when it was within a 10 cm radius of the starting sphere and disappeared after participants had successfully aligned the controller. The starting sphere then turned green and the target line was visible for 500 ms. After the target line had disappeared, participants were instructed to move the controller to the endpoint of the target line that was nearest to them and draw it. If the controller left the starting sphere too early, the starting sphere turned red, and participants had to move the controller back to the starting sphere. In this way, we ensured that participants were copying a remembered line, so that visuo-proprioceptive matching errors^33^ would not affect task performance. To start and end the drawing, participants pressed and released the trigger of the controller, respectively. Participants knew that a drawing movement was registered when the controller vibrated lightly. Once the trigger was released, visual feedback on the trial number and, depending on the experimental phase, performance feedback was provided. After a 300 ms inter-trial interval, the participant could initiate the next trial by aligning the controller with the starting sphere.

In each experiment, there were five phases of 50 trials each: a baseline phase, three learning phases, and a retention phase (Fig. 8b). In the baseline and retention phases, no performance feedback was given. In the learning phase, binary reward feedback (Fig. 8c) was provided according to a reward criterion with an adaptive and a constant component. The adaptive component was used to ensure that participants retained the opportunity to score points when they improved their performance and rewarded trials based on a moving median of the last five trials. Using an adaptive reward criterion ensured that participants would retain the opportunity for being rewarded, even when performance drifts off. This enhances learning relative to using a constant criterion only^5^. The constant component was used to ensure that once participants had achieved good performance they would be reinforced, including when an error was slightly larger than the previous errors and rewarded trials within a fixed range. Whereas the adaptive component depended on the chosen factor, the constant component depended on the multidimensional task. This was done because, in a previous study^14^, we experienced that differences in learning between conditions could be attributed to differences in the constant criterion when this was based on the chosen factor. In the third learning phase, all participants performed the multidimensional task. In the first and second learning phase, task complexity was reduced for the factorized groups. Participants in the Integrated Always group performed the multidimensional task throughout the whole experiment (Fig. 8b).

### Procedure

Before the experiment, we measured eye distance with a ruler and stereovision with the StereoFly test. Next, participants received written and verbal instructions on the experimental task. After the instructions, participants put on the virtual reality headset. We let participants familiarize themselves with the drawing task in four practice trials in which a different target trajectory was shown and, in contrast to the experiment, full visual feedback on the drawn trajectory was provided. After that, the experiment started. As motivation may influence how participants learn from score rewards, motivation was assessed after the baseline phase and following each learning phase using a Quick Motivation Index^22^, in which participants responded orally to the following two questions that were posed by the experimenter using a 1–10 numerical scale: “How much did you enjoy the task until now?” and “How motivated are you to continue?” When the drawing task was finished, the participants’ total score was added to a scoreboard and the participant completed an exit interview in which we asked them about handedness, age, sex, height, clarity of the instructions, explicit knowledge of performance errors and strategies to score points.

### Data analysis

To discard trials on which the trigger was released too early, we excluded trials on which the length of the drawn trajectory was less than 3 cm. This resulted in the exclusion of less than 1% of the data. To determine how much was learnt, we defined the *error level* for each phase: the absolute of the median error in the last 20 trials of that phase. *Learning* within a phase was quantified as the error level in the baseline minus the error level in that phase.

### Statistical tests

Because the error level was an absolute value that causes right-skewed distributions, we used non-parametric tests. We used one-sided tests as we have clear predictions on the direction of the effects. To classify the strength of evidence, we used the Bayesian equivalence of the frequentist tests. Bayesian analyses were performed in JASP^34^. The default Cauchy prior widths (0.707) in JASP were used but we increased the number of samples from 1000 to 10.000. Jeffreys’s evidence categories for the interpretation of Bayes Factor^23^ were used for evaluation of the reported Bayes Factors.

We assessed whether the different factors could be learnt by testing whether the error level in the third learning phase was reduced relative to the baseline phase using one-sided Wilcoxon signed-ranks tests for each group and each factor. We assessed whether learning of a factor (slant, length, curvature) was improved when task complexity was reduced with factorization by testing whether, in the first learning phase, the groups that received factorized feedback on the factor learnt more than the groups who did not receive factorized feedback. For this, we used right-tailed Mann–Whitney U tests. We assessed whether providing factorized feedback improved learning of the multidimensional task by testing whether, in the third learning phase, learning was higher for participants who received factorized feedback than for participants who received integrated feedback using a right-tailed Mann–Whitney U test.

## Experiment 1

### Participants

Seventy-nine participants took part in Experiment 1. They were blindly assigned to one of the four Factorization groups in random order (19 to Slant First, 20 to Length First, 20 to Composite Always, and 20 to Slant First Implicit). After data analysis, and inspired by reviewer comments, we observed that at baseline some participants were already very good at the task. As the composite error left little room for improvement, they were not very well suited to study learning. To include only participants with sufficient room for learning, we excluded participants with a baseline error level in the composite factor smaller than 4 cm (5 from the Slant First group, 2 from the Length First group, and 2 from the Slant First Implicit group). We tested 8 new participants (4 in the Slant First group, 2 in the Length First group, and 2 in the Slant First Implicit group). This resulted in a final sample of 78 participants (age 21.0 ± 4.0; 17 male, 58 females; 49 right-handed, 6 left-handed, 4 unregistered handedness).

### Factorized feedback

We provided binary reward feedback by adding 5 points to a cumulative score, playing a ‘ping' sound, and colouring the target line green after a successful trial and doing nothing after a non-successful trial. Participants received no other performance feedback. Whether a trial was rewarded or not was based on a vector between the starting point and endpoint of the drawn line, projected on the sagittal plane (the plane dividing the body into left and right halves; Fig. 8d). The vector was factorized into two factors: slant and length. Between groups, we varied whether these factors were trained sequentially in the first two learning phases—‘factorized’ feedback—or whether we always provided feedback on the composite. The slant feedback was based on vector slant in the sagittal plane. The length feedback was based on vector length. The integrated feedback was based on the vector difference between the drawn vector and the target line. This way, three types of error could be defined (Fig. 8d).

In the third learning phase, all participants received feedback based on the composite error; the feedback in the first two learning phases differed between the groups. Participants in the Length First group performed the first learning phase with feedback based on length, the second learning phase with feedback based on slant. Participants in the Slant First group performed the first learning phase with feedback based on slant, the second learning phase with feedback based on length. Participants in the Composite Always group performed all three learning phases with feedback based on the composite error. The constant component of the reward criterion rewarded trials on which the composite error was smaller than 2 cm. We based the constant component on the composite error in all phases and groups for standardization between groups.

### Procedure

Participants stood behind the starting position, the height of which was scaled to their height (80% of headset height above the floor). They were instructed that they should try to match the target line as accurately as possible with the movement of the controller. Participants in the Slant First group were told that their scores would first depend on slant, next on length, and finally on the combination of the two. The Length First group was told that their scores would first depend on length, next on slant, and finally on the combination of the two. Participants in the Composite Always group were told that their scores would depend on the combination of slant and length. Illustrations were used to inform the participants how slant and length were defined. Participants in the Slant First Implicit group did not receive explicit information on performance-relevance. They were told that they had performed well when the target coloured green and points were scored.

## Experiment 2

### Participants

Sixty participants took part in Experiment 2 (age 20.2 ± 2.0; 7 male, 53 females; 54 right-handed, 4 left-handed, 1 unregistered handedness). They were blindly assigned to one of the three Factorization groups in a random order (21 to Slant First, 19 to Curvature First, 20 to Integrated Always.

### Factorization

The drawing task was factorized in three factors: slant, curvature, and the integrated factor (Fig. 8e). The slant factor was calculated as in Experiment 1. The curvature factor was calculated using the point midway the movement and the directional vector between the starting point and endpoint of the trajectory. We calculated the distance from the point midway the movement to the directional vector and divided this value by the length of the directional vector. The integrated error represented the total spatial difference between the target and the drawn trajectory. This error was calculated by resampling both trajectories to 50 points, equally sampled in space and taking the average distance between the samples of the target and drawn trajectory.

In the third learning phase, all participants received feedback on the integrated error; the feedback in the first two learning phases differed between the groups. Participants in the Slant First group performed the first learning phase with feedback based on slant and the second learning phase with feedback based on curvature. Participants in the Curvature First group performed the first learning phase with feedback based on curvature and the second learning phase with feedback based on slant. Participants in the Integrated Always group performed all three learning phases with feedback based on the integrated error. The constant component of the reward criterion rewarded trials on which the integrated error was smaller than 1 cm.

### Procedure

Based on our experience in Experiment 1, we made a few changes to the methods. First, participants in Experiment 2 performed the task seated to prevent position changes during the experiment in a natural way. Second, participants in Experiment 2 could make their drawing movement while the target remained visible. This was done because some participants in Experiment 1 had trouble delaying their copying movement until the moment the target disappeared. Third, whereas participants in Experiment 1 rated their enjoyment and motivation to continue on the QMI orally, participants in Experiment 2 used a slider rendered in virtual reality to rate their motivation on a continuous scale from ‘not at all’ to ‘very much’. Unfortunately, this appeared to be ineffective. As participants tended to use only the extremes of the slider, QMI scores in Experiment 2 are not reported.

## Data Availability

The datasets generated and analysed during the current study and the MatLab code used in the analysis are available in the Open Science Foundation repository, [https://osf.io/vsdt5/].
